# Glycoprofile Comparison of the SARS-CoV-2 Spike Proteins Expressed in CHO and HEK Cell Lines

**DOI:** 10.1007/s12033-024-01288-2

**Published:** 2024-10-01

**Authors:** Helen L. Wright, Caroline Evans, Philip J. Jackson, David C. James, Kang Lan Tee, Tuck Seng Wong, Mark J. Dickman, Jagroop Pandhal

**Affiliations:** 1 School of Chemical, Materials and Biological Engineering, University of Sheffield, Mappin St., Sheffield, S1 3JD UK; 2 School of Biosciences, University of Sheffield, Western Bank, Sheffield, S10 2TN UK

**Keywords:** COVID-19, SARS-CoV-2, *N*-glycosylation, CHO Cells, HEK cells

## Abstract

Coronavirus SARS-CoV-2 spike protein remains a key focus of research due to a continued need for diagnostic and therapeutic tools to monitor and respond to new variants. Glycosylation of the spike protein is critical for the protein’s functions in viral attachment and host cell entry. For scalable and cost-effective production of the spike protein, expression system-driven divergence in glycosylation patterns on recombinant spike proteins needs to be fully understood. This study assessed the *N*-glycosylation profiles of a full-length trimeric spike protein expressed in either Human Embryonic Kidney (HEK Expi293F) or Chinese Hamster Ovary (CHO-S) cells. Glycopeptide analysis was performed using a tandem mass spectrometry workflow and BioPharma ${\hbox {Finder}}^{\textrm{TM}}$ incorporating HEK and CHO glycan databases for protein characterisation. The results outline important differences in the variety and types of *N*-glycan generated by the two cell lines across the 22 known *N*-glycosylation sites of the spike protein. A notable increase in terminal sialylation, as well as the presence of the potentially immunogenic *N*-glycolylneuraminic acid at a functionally key *N*-glycosylation site, was observed in the CHO-S derived spike protein. With the potential for the relatively vast and more complex CHO glycan repertoire (182 glycans relative to 39 human glycans) to produce functional implications with CHO-S expressed spike protein, this study adds valuable knowledge to aid Quality by Design approaches and enable Multi Attribute Monitoring of specific *N*-glycosylation sites for proteoform analyses. This can further inform antigen development with future variants in order to devise updated diagnostic tests and therapeutic vaccine designs.

**Supplementary Information:**

The online version contains supplementary material available at 10.1007/s12033-024-01288-2.

## Introduction

At the end of 2019 a new virus of the coronaviridae family emerged in the human population of Wuhan, China. Severe acute respiratory syndrome coronavirus 2 (SARS-CoV-2) was identified in December 2019 as the causative agent of what became the global pandemic known as COVID-19 [1–3]. By the start of 2024, SARS-CoV-2 had been the cause of 774,771,942 confirmed cases of COVID-19 worldwide, resulting in the deaths of 7,035,337 people across the globe (World Health Organisation, February 25th, 2024).

Host cell infiltration by the coronavirus is mediated by the trimeric spike protein, which protrudes from the surface of the viral capsid and binds to the angiotensin-converting enzyme receptor (hACE2 receptor) on host respiratory cells [4]. The spike protein exhibits high levels of glycosylation and its receptor binding domain (RBD) is highly immunogenic, making it a key target for many neutralising antibodies [5, 6]. The trimeric SARS-CoV-2 spike protein comprises two functional subunits S1 and S2; S1 mediates binding of the virus to the host hACE2 receptor and S2 facilitates viral entry into host cells through fusion with the cell membrane. The spike protein exhibits 22 *N*-linked glycosylation sites on each monomer (66 total per trimer) [7], and up to 30 potential O-linked glycosylation sites have also been discovered thus far [8, 9]. Additionally, several of these potential O-glycosites were found to occur close to the *N*-glycosylated asparagine residues [8]. Glycans on viral surface proteins can facilitate immune evasion by using host synthesised glycans to ’shield’ their own non-human protein epitopes from binding and neutralisation by host antibodies [7]. Glycosylation on both the viral spike protein and its target hACE2 receptor have the ability to impact their interaction and binding. High levels of glycosylation can be found at the interface of these two proteins [10], and mannose residues within these glycans were found to interact with cell surface attachment factors [11, 12]. Studies comparing glycosylation of different SARS-CoV-2 variants have found that the presence or absence of sialylation on RBD glycans has a regulatory role on the binding affinity of the spike protein [13, 14]. Furthermore, the anti-SARS-CoV-2 neutralising antibody S309 was able to reduce the binding affinity of several different RBD glycoforms, further implicating the role of glycans in RBD function [13]. *N*-glycan processing has been found to be conserved at most *N*-glycosylation sites across different variants, however some variants exhibit a higher proportion of under-processed *N*-glycans at sites N165, N343 and N616 [15]. These sites have previously been implicated as key regulators of the interaction between the spike protein and the hACE2 receptor [16, 17]. Sequence analysis of mutations in key variants of concern (VoCs) also revealed the creation of two new *N*-glycosites, N20 and N188 in the recent Gamma variant, as well as the loss of at least one *N*-site in the Delta, Omicron and Lambda variants [15]. Increases in high mannose content were also observed in the Gamma and Delta variants, and these variants were also shown to exhibit reduced core-fucosylation and sialylation levels [18]. The spike protein became a focal target for diagnostic, treatment and vaccine development, and thus it is vital to fully understand the protein’s glycosylation profile in order to identify aspects of the SARS-CoV-2 biology that may influence future vaccine design [17, 19–21].

With the current industry desire to find suitable producing platforms that exhibit high reliability, high efficiency and low production costs, investigation of the variations in post translational modifications produced by different expression systems must also be considered of utmost importance. Mammalian cells currently dominate as a biomanufacturing platform due to their unrivalled ability to produce larger protein products with complex post translational modification. In particular they have the ability to produce human-compatible glycosylation, despite requiring more complicated, expensive and longer culture processes than platforms such as insect, bacterial, and yeast cells [22, 23]. The spike protein has already seen successful production in a variety of mammalian and non-mammalian cell types, including S*f*9 insect cells and *Chlamydomonas* algae [24–26]. However, differences in the glycosylation machinery exhibited by different cell types can produce vastly altered glycoforms of the spike protein, which may impact properties such as circulating half-life, binding affinity, thermal stability and potency for immune response [27–30]. Indeed, spike protein expressed in mammalian expression systems has already been shown to elicit higher titres of neutralising antibodies in mice, compared to an insect-derived equivalent [27]. It was proposed that this reduced immunogenicity is potentially due to the lack of human-like sialylation produced by the commonly used insect cell line TN-5B1-4. There has been limited investigation into the glycosylation produced on spike protein by the non-mammalian insect platforms [25], and glycosylation on algae-produced spike protein is yet to be reported.

**Fig. 1:**
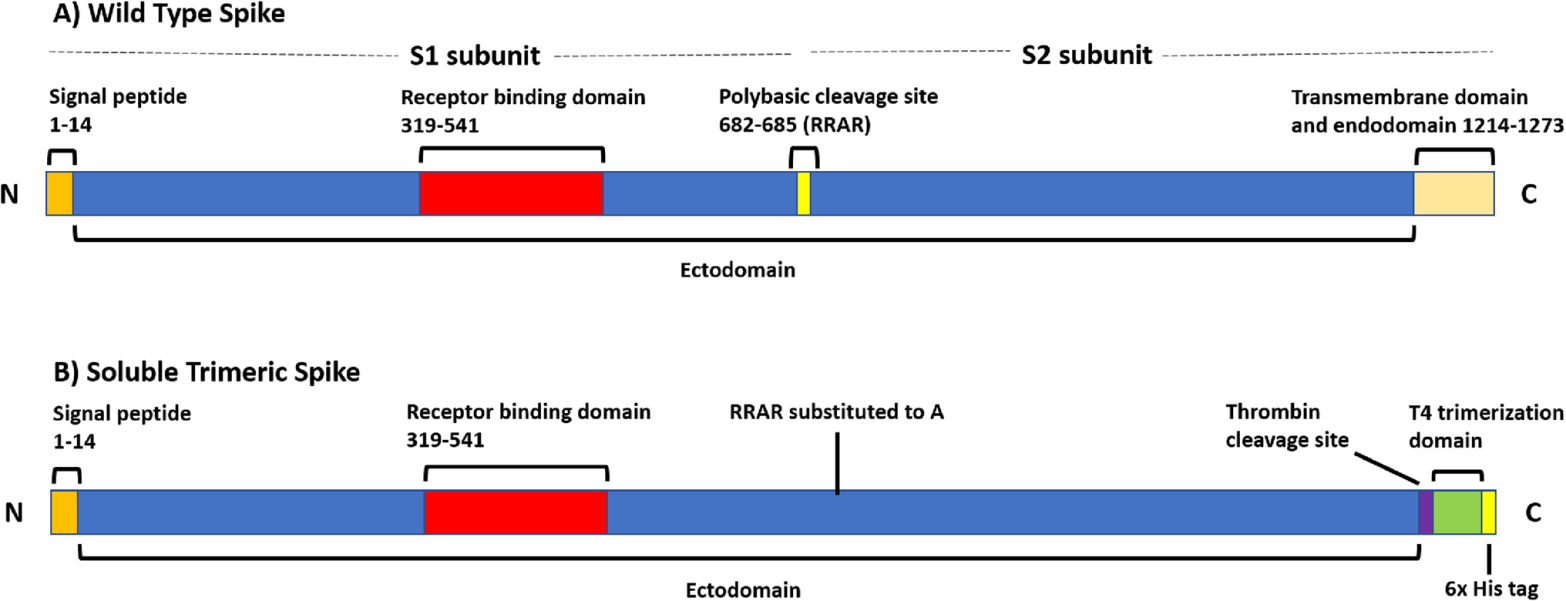
Schematic of the soluble trimeric spike protein construct expressed in Chinese Hamster Ovary (CHO-S) and Human Embryonic Kidney (Expi293F) cells. A His tagged, stabilised construct was used to produce the full length, glycosylated spike protein. The soluble trimeric spike construct contains multiple stabilisation modifications: the polybasic (furin) cleavage site is substituted with a single alanine (**A**) residue; two stabilising mutations K986P and V987P were introduced; a thrombin cleavage site at P1213, T4 bacteriophage trimerisation domain and a 6x His tag were fused to the truncated C terminal transmembrane and endodomain. **A** Wild type, and **B** soluble trimeric spike construct

In 2020 an interdisciplinary team at the University of Sheffield successfully expressed full length soluble, trimeric SARS-CoV-2 spike protein for use as an antigen for ELISA-based serological detection of immunoglobulin G antibodies against SARS-CoV-2 in clinical samples [31, 32]. For the expression system, Chinese Hamster Ovary (CHO-S) cells were chosen to biomanufacture the spike protein due to the platform’s human compatible glycosylation capability and ease of biomanufacturing scale up [31]. For decades CHO cells have dominated as the cell manufacturing platform of choice for recombinant biotherapeutics [23]. Between 2018 and 2022, 89% of United States and/or European Union approved recombinant products manufactured in mammalian cells were produced in CHO lines [23]. Johari et al. also expressed the spike protein construct in a Human Embryonic Kidney (HEK) Expi293F cell line, so that performance of the CHO-S derived spike in ELISA testing could be compared against the human cell derived equivalent, which has already been approved for use in serological COVID-19 antibody testing by the United States FDA [19, 31]. For improved stability in production, this soluble trimeric construct carries two stabilising mutations K986P and V987P and substitutes the furin cleavage site with a single alanine (A) residue, relative to the wild type protein (Fig. 1). Additionally, the C-terminal transmembrane and endodomain is substituted with a P1213 thrombin cleavage site, a T4 trimerisation domain and a 6x His tag for purification [19].

In this work the CHO-S derived, His tagged trimeric protein was glycoprofiled and compared to the same construct expressed by the human cell line (HEK Expi293F). Despite its ability for fully human-type processing and modification of therapeutic proteins, HEK cells have fallen out of favour as a manufacturing platform in recent years, with only 1 approved recombinant product made by these cells between 2018 and 2022 [23]. The monopoly now held by CHO production platforms is largely due to the relative ease of cultivation and high titres of antibody production (3–8 g/L) seen at production level scale [33]. This popularity of CHO cells has persisted despite differences in its glycosylation profile being identified as far back as the mid-2000s [22]. The purpose of this study was to further investigate the degree of human compatibility of the CHO-S cell line glycoprofile by comparison against HEK Expi293F as a ‘benchmark’ for human glycosylation patterns on recombinant spike protein. This information could then be used to direct the future development of the CHO-S derived spike protein for clinical and diagnostic studies.

It has been found in several studies that the *N*-glycan repertoire of some CHO cell lines, while similar to human cells, does exhibit differences in complexity, composition and variety of glycan motifs [28, 34–36]. This is due to variations in the collection of enzymes and transporters used by different cell lines for the mammalian glycosylation process [34]. These differences in the final glycosylation pattern of manufactured antigens could have a noticeable impact on their efficacy during vaccine development and serological testing [36–38]. Despite this, CHO derived recombinant proteins remain one of the closest to human-like in terms of the post translational modifications they possess, such as glycosylation and phosphorylation [39]. It is likely they will remain the most commonly used mammalian expression system for the foreseeable future, due to several desirable qualities. These cells have the ability to grow to high cell densities in serum free media; the preferred method for large scale bioreactor culture, with CHO cell culture now possible at over 10,000 L scale in stirred tank bioreactors [39, 40]. As a result of decades of cell line development through cell and vector engineering, and culture process optimisation, these cells can now achieve higher than 10 g/L protein production capable of meeting the high market demand for a wide range of biopharmaceutical active molecules [40]. CHO cells have been successfully used to produce virus-like particles (VLPs) presenting the spike protein that were proven to raise neutralising antibodies and provide immunity to SARS-CoV-2 infection in rodents [41]. These CHO-derived VLPs show promise for further development as part of a cost-effective, alternative spike based VLP vaccine similar to the currently available Covifenz® (Medicago) vaccine made in a plant system [41]. However, a more detailed understanding of CHO glycosylation on the spike protein is necessary to determine the effect of expression system-dependent glycan variation on the immunogenicity of this antigen for vaccine development.

## Materials and Methods

All reagents were purchased from Sigma-Aldrich, unless otherwise stated.

### Transient Expression of Spike Proteins in CHO-S and HEK Expi293F Cells

Spike protein samples derived from CHO-S and HEK Expi293F were provided by Johari et al. [31]. Method details for the expression and purification of the proteins have been provided here for reference.

HEK Expi293F cells were cultured in Expi293 Expression medium (Thermo Fisher Scientific) in Erlenmeyer flasks maintained at $37\,^{\circ }{\hbox {C}}$, 125 rpm with 8% ${\hbox {CO}}_{2}$ and 85% humidity. CHO-S clonal isolate cells (C1-80) [42] were cultured in CD CHO medium (Thermo Fisher Scientific) supplemented with 8 mM L-glutamine. Cultures were maintained at $37\,^{\circ }{\hbox {C}}$, 140 rpm with 5% ${\hbox {CO}}_{2}$ and 85% humidity. Cells were seeded at 2 $\times$ 10^5^ viable cells/mL and were sub-cultured every 3–4 days. Cell viability and VCD were measured using the Vi-CELL XR (Beckman Coulter). The IVCD was calculated as described in Johari et al. [31].

pCAGGS plasmids encoding the stabilised full-length, soluble SARS-CoV-2 spike protein trimer were provided by the Krammer Laboratory (Icahn School of Medicine at Mount Sinai) [19]. These plasmids contain a CAG promoter, glutamine synthetase gene and CMV enhancer. Plasmids were amplified and purified using QIAGEN Plasmid Plus kit (Qiagen).

For Expi293F transfection, cells were grown to $1.75\times 10$^6^ cells/mL, centrifuged and resuspended at $3.5 \times 10$^6^ cells/mL, followed by sequential addition of $0.85\;\upmu$g of DNA and $2.55\;\upmu$L of PEI MAX per 1 million cells (both pre-diluted in $10\;\upmu$L of 150 mM NaCl). At 24 h post-transfection, the cells were diluted 2$\times$ by adding fresh medium, and the temperature was shifted to $32\, ^{\circ }{\hbox {C}}$ for 8 days of cultivation.

For CHO-S transfection, cells were seeded one day before transfection and grown to 1.5 $\times$ 10^6^ cells/mL. For every 1.5 $\times$ 10^6^ cells, $1.3\;\upmu$g of DNA and $4.55\;\upmu$L of PEI MAX (each pre-diluted in $15\;\upmu$L of 150 mM NaCl) were incubated at room temperature (RT) for 2 min before being added into the culture. The temperature was shifted to $32\, ^{\circ }{\hbox {C}}$ at 4 h post-transfection for 10 days of fed-batch cultivation, with 5% v/v CHO CD EfficientFeed B (Thermo Fisher Scientific) every 2 days.

### Recombinant Protein Purification

Spike protein was harvested by centrifugation at 3000 $\times$ *g* for 20 min at $4\, ^{\circ }{\hbox {C}}$ and the supernatant filtered through a $0.22\;\upmu$m filter. The spike protein was purified using an ÄKTA Pure system (Cytiva) with a 5 mL HisTrap HP column (Cytiva). The column was first washed with 5 column volumes (CVs) of elution buffer (50 mM sodium phosphate, 300 mM NaCl, 250 mM imidazole, pH 8.0), and subsequently equilibrated with 5 CVs of binding buffer (50 mM sodium phosphate, 300 mM NaCl, 10 mM imidazole, pH 8.0). After sample loading, the column was washed with three times 5 CVs of binding buffer, 5 CVs of 4.5% v/v elution buffer, and 10 CVs of 9% v/v elution buffer. The protein was eluted using 100% v/v elution buffer. Protein fractions were pooled and buffer exchanged into storage buffer (20 mM Tris, 200 mM NaCl, 10% v/v glycerol, pH 8.0) using a PD-10 desalting column (Cytiva). Data pertaining to the expression yield and purification of the proteins can be found in Johari et al. [31].

### SDS PAGE

Protein fractions were run on a ${\hbox {NuPAGE}}^{\textrm{TM}}$ 4–12%, Bis–Tris, 1 mm, 12-well mini protein gel (Thermo Fisher Scientific) using NuPAGE^TM^ MES SDS running buffer (Thermo Fisher Scientific) at a constant voltage of 200 V for 40 min. For visualisation, the gel was stained using InstantBlue^TM^ (Expedeon).

### Protease Digestion and Peptide Extraction

Protein digestion and preparation for analysis by reverse-phase liquid chromatography–mass spectrometry (RPLC–MS) was performed as previously described by Johari et al. [31]. Briefly, protein samples were first concentrated using YM-30 spin filters. Two $10\;\upmu$g preparations in 50 mM ammonium bicarbonate (ABC) were reduced using 5 mM tris(2-carboxyethyl)phosphine–HCl at $37\, ^{\circ }{\hbox {C}}$ for 30 min. S-alkylation was performed by the addition of $1\;\upmu$L 100 mM methyl methanethiosulfonate in isopropanol. Proteins were then subject to proteolytic digestion using 0.02% ProteaseMax surfactant in 50 mM ABC and $0.4\;\upmu$g trypsin (Promega) to one preparation and $0.4\;\upmu$g GluC (Promega) to the other followed by incubation at $37 \,^{\circ }{\hbox {C}}$ for 16 h. Proteolysis was stopped and the surfactant hydrolysed using 0.5% trifluoroacetic acid (TFA). The peptides were then desalted using C$_{18}$ spin columns (Thermo Fisher Scientific) and diluted to 250 ng/$\upmu$L in loading solvent.

### Protein Identification by Mass Spectrometry

LC–MS/MS was performed and analysed by nano-flow liquid chromatography (U3000 RSLCnano, Thermo Scientific) coupled to a hybrid quadrupole-orbitrap mass spectrometer (Q Exactive HF, Thermo Scientific). Peptides were separated on an Easy-Spray C$_{18}$ column (75 $\upmu$m $\times$ 50 cm) using a 2-step gradient from 97% solvent A (0.1% formic acid in water) to 10% solvent B (0.1% formic acid in 80% acetonitrile) over 5 min then 10% to 50% B over 75 min at 300 nL/min. The mass spectrometer was programmed for data dependent acquisition with 5 product ion scans (resolution 30,000, automatic gain control 2e5, maximum injection time 250 ms, isolation window 2 Th, stepped collision energy 20, 30, 40 intensity threshold 2e3, fixed first mass 100) per full MS scan (resolution 120,000, automatic gain control 1e6, maximum injection time 100 ms) with a 20 s dynamic exclusion time.

### Peptide and *N*-Glycopeptide Analysis

Glycopeptide analysis was performed using the peptide-mapping tool of BioPharma ${\hbox {Finder}}^{\textrm{TM}}$ 4.0 Mass Informatics Platform for Biopharmaceutical Characterisation (Thermo Fisher Scientific). Glycopeptide identification and quantitation was performed using the integrated human and CHO *N*-glycan databases for the HEK Expi293F and CHO-S samples, respectively, specifying either trypsin or Glu-C cleavage. Calculated sequence coverage was as follows: HEK Expi293F trypsin 66.2%, GluC 64.5%; CHO-S trypsin 77.5%, GluC 69.5%. The glycan databases for HEK and CHO cells are composed of 39 and 182 *N*-glycan structures, respectively. Glycopeptide and peptide assignments required Full MS and MS/MS data, mass deviation of 5 ppm and minimum confidence of 95%. Assignments with sodium or potassium adducts, or corresponding to nonspecific protease activity, gas phase-generated ions or unspecified modifications were excluded [43]. Quantitative data were calculated from average values of triplicate injections, and data from trypsin and GluC digestion was pooled by manufacturing platform. A breakdown of the glycan and *N*-glycosylation site coverage achieved by each enzyme for HEK Expi293F and CHO-S can be found in the Supplementary Materials (Supplementary Table S3). The relative abundance of the classified glycans was calculated from the mass spectral peak area at MS1 level averaged from three technical replicates. Note that in this data the *N*-glycosylation sites from N706 onwards are $-3$ relative to the wild type SARS-CoV-2 spike protein, due to the substitution of PPAR residues 682–868 to a single A residue (as seen in Fig. 1). For the purpose of comparison these sites are annotated according the equivalent wild-type site in the figures and tables presented.

## Results and Discussion

### *N*-Glycan Sites and Glycan Identification

The BioPharma ${\hbox {Finder}}^{\textrm{TM}}$ software successfully identified glycan residues present at all 22 known *N*-linked glycosites present on the spike protein expressed in both the CHO-S and HEK Expi293F cell platforms. After stricter confidence filtering was applied to the glycopeptide assignments (see Materials and Methods Sect. 2.6), glycosylation states found at N706 were omitted in the final overall and site-specific analysis. Details of the glycan entries for this *N*-glycan site can be found in Supplementary Table S5. Pooling of data generated by trypsin and GluC digestion allowed for peptide coverage of all 22 sites, and identification of additional glycan species that were not discovered using either enzyme alone. Differences in *N*-glycosylation data generated by trypsin and GluC have been observed previously on another CHO-derived spike protein [44]. Further details of the respective trypsin and GluC data generated in this study can be found in Supplementary Table S3.

The CHO-S expressed spike construct exhibited a greater number and variety of glycans, compared with the HEK Expi293F expressed construct: in total 143 individual glycans were identified on the CHO-S expressed protein and only 37 on the HEK Expi293F expressed equivalent. Overlap of glycans discovered in both species can be seen in the Supplementary Materials (Supplementary Table S4). There are considerably more species than has been previously described on a CHO-expressed spike protein [45, 46]. Previous studies have shown that CHO cell lines produce a greater variety of glycans, while HEK cell lines tend to produce fewer, but more complex species [28, 34, 35].

The comparatively vast glycan library available with CHO cell glycosylation means that many more combinations of glycans are possible at each of the 22 *N*-glycosites on the CHO expressed spike protein. This could potentially enhance the shielding effect of the spike protein making immunodetection of the antigen sites more difficult [7, 17]. Again, this has implications for raising antibody-based therapies against this spike construct: creating effective antibodies against such a variety of glycopatterns could be considerably more difficult, however it could then produce polyclonal therapies that are effective against a number of different strains of the SARS-CoV-2 virus. Spike protein that exhibits a higher degree and complexity of glycosylation has been shown to produce higher titres of neutralising antibodies than using partially glycosylated trimers [27]. Use of a combination of spike proteins exhibiting a variety of glycosylation, in prime-boost-boost immunisation procedures, also produced a stronger immune response than using homogeneously glycosylated spike protein. Furthermore, partially glycosylated mammalian and insect-derived spike protein produced a weaker reaction with monoclonal antibodies targeting various domains of the recombinant antigen [27].

Ideally a spike protein antigen to be used for vaccine development needs to exhibit human-type glycosylation patterns, since the host cell machinery is ‘hijacked’ by the virus during infection to produce subsequent viral proteins. If these recombinant proteins are to be recognised by the host immune system or any immunotherapeutic drugs, the pattern of glycosylation should match the host system as closely as possible. Deng et al. proposed that patient antibodies produced by infection with SARS-CoV-2 could be glycan-dependent. Changes in spike protein glycosylation through either variant mutations or cell manufacturing of recombinant vaccines could result in the absence of native antigenic regions and prevent immune recognition in subsequent infections [27]. However, a balance is needed to avoid production of densely glycosylated vaccine antigens that could contribute to shielding of immunogenic epitopes and aid immune evasion [47].

### Classification by Glycan Type

**Fig. 2:**
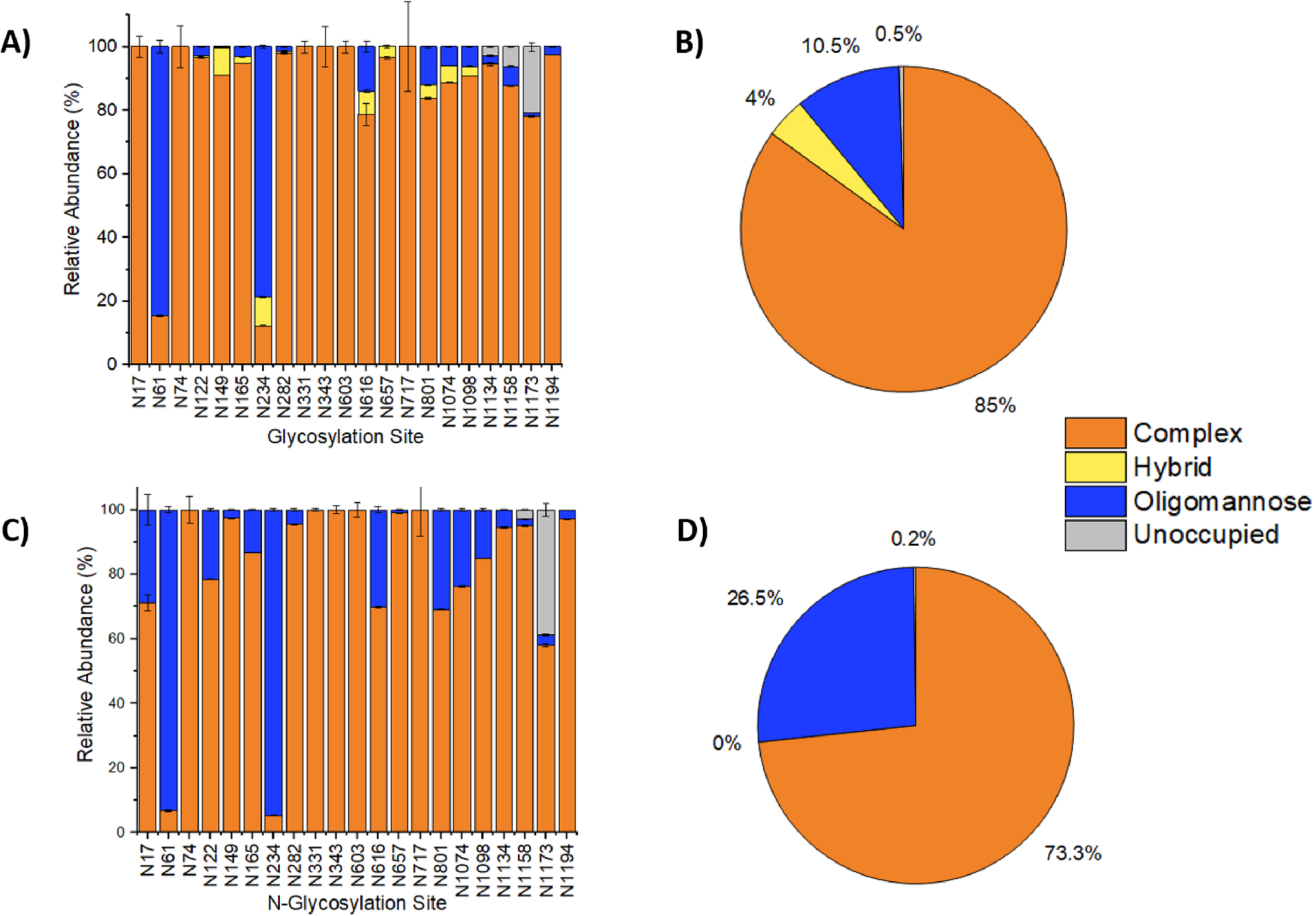
Relative abundance of N glycan classification for CHO-S and HEK Expi293F expressed spike protein. Proportion of complex, oligomannose, hybrid and unoccupied sites discovered at each *N*-glycosylation site (**A**, **C**), and across the 21 high confidence *N*-glycosylation sites (**B**, **D**) for CHO-S and HEK Expi293F

Identified glycans were organised into the three standard categories of glycan species; complex, oligomannose and hybrid [48]. The relative abundance of each category was calculated for each *N*-glycosite. Instances where a site was found to be unoccupied were also recorded and the relative abundance of the unoccupied state was also calculated. Finally, the overall percentage of each glycan category (or unoccupied site) found across the entire protein was calculated for CHO-S and HEK Expi293F. The results of this classification process are presented in Fig. 2.

The relative abundance of complex and oligomannose glycans at each *N*-linked site followed a similar pattern for both CHO-S and HEK Expi293F cells (Fig. 2A, B). Specific sites in both cells showed a predominance of one or the other type; for example N61 and N234 showed a predominance of oligomannose type glycans in both cell types, whereas all other sites exhibit complex type glycans as the most abundant category. Predominating levels of oligomannose glycans have been observed previously at these two sites, but notably has also been observed at N603 and N709 in other studies [7, 25, 41]. N61 has also been observed to be mostly oligomannose type in S*f*9 insect cells, though this platform exhibited a prevalence of truncated glycans overall, and only low levels of complex type glycans [25]. In this study, a shift in oligomannose content was observed at two key sites for receptor binding, N165 and N616, in the CHO-S protein compared to the equivalent sites in the HEK Expi293F protein. A reduction in oligomannose content at these sites could result in reduced binding affinity due to steric hindrance caused by the presence of higher complexity glycans instead [46]. Instances where no glycans were found (unoccupied sites) were exclusive to later *N*-linked sites close to the thrombin cleavage site and trimerisation domain; N1134, N1158 and N1173 in CHO-S cells, N1158 and N1173 for HEK Expi293F. To the authors’ best knowledge there are no known biological functions associated with these *N*-glycosites at the C-terminal end of the spike protein. Hybrid type glycans were only detected in the CHO-S derived spike protein in this study, but have been found in HEK293 expressed spike protein by other studies [7, 25, 45]. Our data corroborates the presence of low amounts of hybrid type glycans at N165, N801 and N1098 shown by Alpuche-Lazcano et al. on soluble spike protein and spike-presenting VLPs produced in CHO^2353^ clone cells (CHO-C2), grown in a proprietary media formulation under similar cultivation conditions [41]. However, the distribution of hybrid glycans at other sites differs between the two studies. Notably, some of the higher proportions of hybrid glycans were found at N717 on both the soluble spike trimers and S-VLPs from the CHO-DXB11 derived clonal cell line, whereas complex type glycans made up the entirety of glycans found in our data for this site from the widely used, stable CHO-S line [41].

Overall, both cell types show complex glycans to be the predominant type, making up 83.0% of glycans found across all sites in CHO-S and 73.3% of glycans in HEK Expi293F (Fig. 2C, D). This is consistent with existing glycoprofiling studies of HEK and CHO derived spike protein [7, 25, 45, 46, 49, 50]. Oligomannose made up the larger portion of the remaining glycan types found, though a lower abundance of oligomannose glycans was found in CHO-S than in HEK Expi293F (10.5% in CHO-S vs. 26.5% in HEK Expi293F). This change to the proportion of oligomannose glycans across the whole protein produced by the CHO-S cell line again differentiates this recombinant protein from the HEK Expi293F produced equivalent and it is likely that this could further impact on factors such as antibody binding kinetics or serum clearance of the protein. Hybrid type glycans accounted for 4% of the glycans found in CHO-S derived spike protein. In this study, the presence of hybrid type glycans was not observed on the same construct expressed by the HEK Expi293F cells, but has been reported on HEK-derived spike protein in other studies [7, 25]. Both cell types exhibited similarly low instances of unoccupied (unglycosylated) sites (0.5% for CHO-S and 0.2% for HEK Expi293F).

### Antennary Structure of Glycans

The complex *N*-glycans at all sites found in CHO-S and HEK Expi293F were then categorised according to their branching structure; glycans consisting of a single branch of sugar moieties (underprocessed/incomplete glycans) or containing two to four branches. The number and structure of branches on glycan motifs is commonly used as a measure of the complexity of a protein’s glycoprofile.

**Fig. 3:**
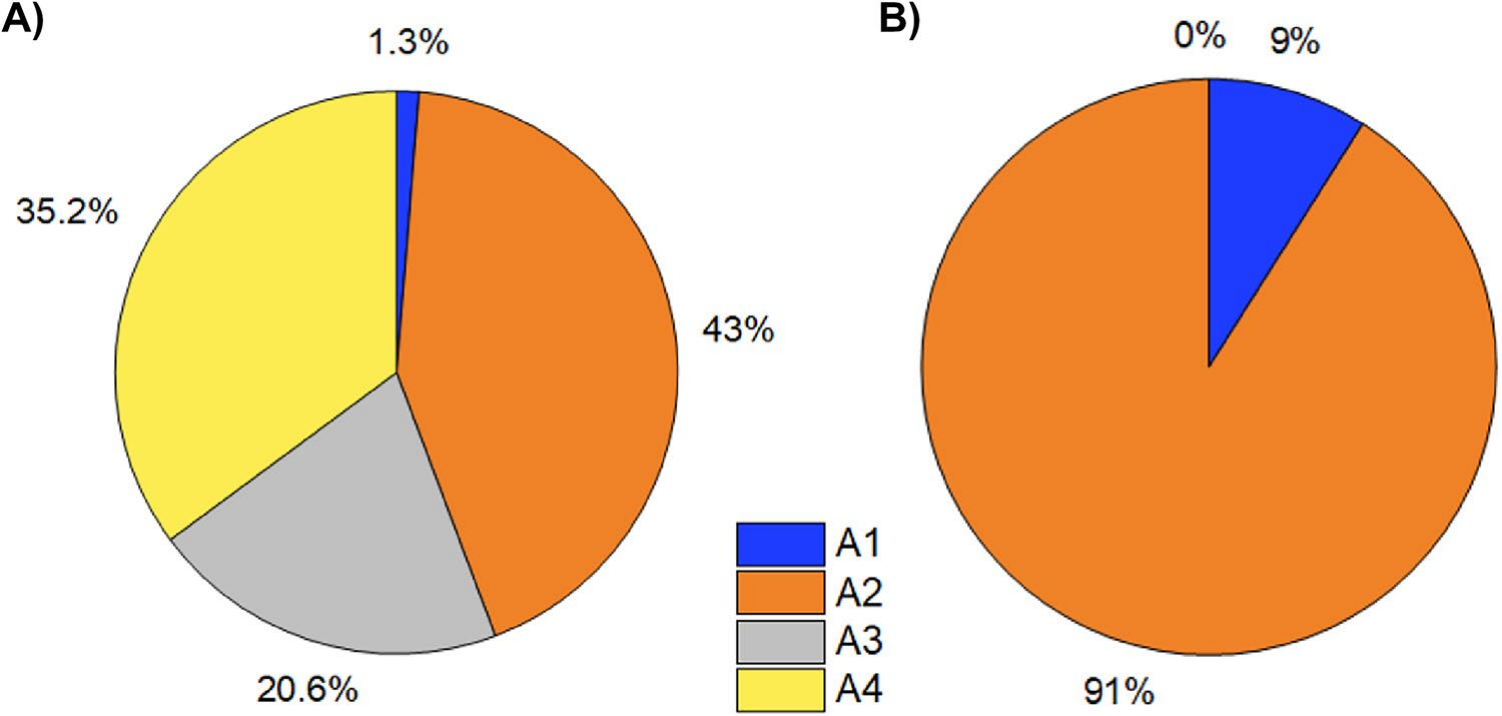
Antennary structure of complex glycans from **A** CHO-S and **B** HEK Expi293F expressed spike protein. Glycans were classified according to number of branches; *A1* single branch/underprocessed glycans, *A2* biantennary glycans, *A3* triantennary glycans, *A4* quadantennary glycans

Figure 3 demonstrates that the spike protein expressed in HEK Expi293 showed predominantly biantennary structure glycans (85–90%), with a small portion of single branch (underprocessed) glycans (10–15%). The CHO-S expressed protein also showed predominance of biantennary glycans (35–40%), but a much smaller proportion of uniantennary glycans (3–8%) than its HEK Expi293F expressed equivalent. This is consistent with existing literature where biantennary glycans are found to be the principle glycan type in both of these cell expression systems [28, 34, 41]. Previous studies of spike protein expressed in HEK293 cells have also reported biantennary glycans to be the highest abundance complex type glycans [7, 46]. Spike protein expressed in CHO-S cells also exhibited the presence of higher branching species (tri- and quadantennary), which were not found in the HEK Expi293F spike protein. However, these glycans have been found on HEK expressed spike protein by other studies [7, 41, 46]. In our data, highly branched glycans made up a considerable proportion of the glycoprofile of the CHO-S expressed protein (21–24% triantennary and 31–38% quadantennary). These levels of tri- and quadantennary glycans are highly comparable to that found previously by Huang et al. on another CHO expressed spike protein [46].

### Presence and Distribution of Core Fucose and Terminal Sialic Acid Residues

**Fig. 4:**
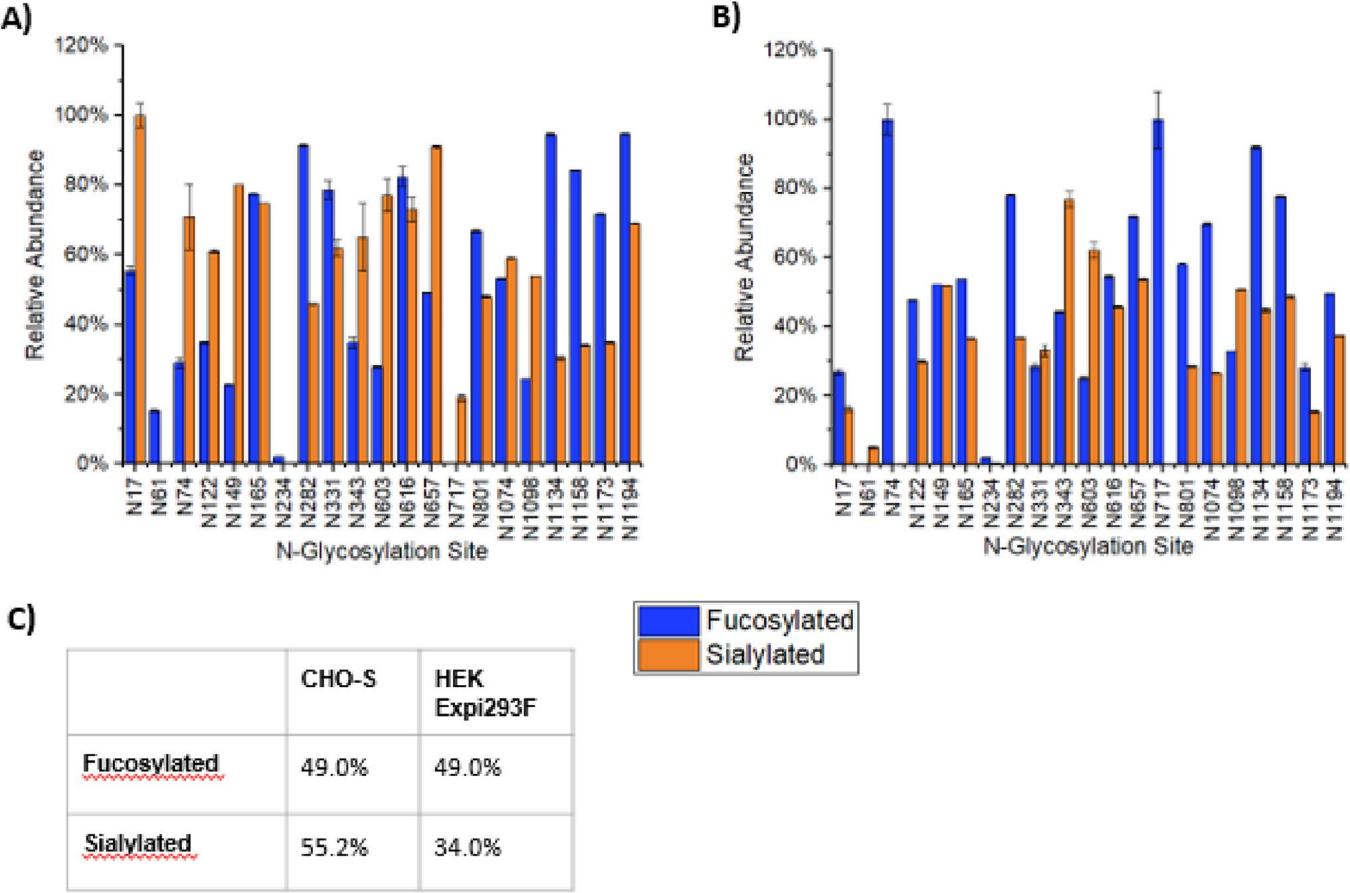
Distribution of core fucosylated and sialylated glycans across the 21 high confidence *N*-glycosylation sites found in **A** CHO-S derived spike protein and **B** HEK Expi293F derived spike protein. The overall proportion of fucosylated and sialylated glycans across all sites is shown in **C**

Identified glycan species were then categorised according to the presence or absence of a core fucose residue and/or terminal sialic acid residues. From this, the relative abundance of core fucose and terminal sialic acid was determined for each *N*-glycosite, as well as overall across all glycosites for both CHO-S and HEK Expi293F (Fig. 4).

Overall levels of fucosylation were found to be equivalent in CHO-S and HEK Expi293F derived spike protein (49%, Fig. 4C), though the distribution of fucosylated glycans differed at individual *N*-sites (Fig. 4A, B). This is consistent with Watanabe et al., who reported spike fucosylation levels to be around 50% for HEK293 expressed spike protein [7]. Another study found fucosylation levels on the S1 subunit to be similar between HEK and CHO, albeit at a slightly higher overall percentage (60%) [46]. In the present study, N234 exhibited similarly low fucosylation levels in both the HEK Expi293F and CHO-S derived protein. For the CHO-S spike, *N*-glycosylation sites at N282, N616, N1134, N1158 and N1194 were found to contain the highest level of fucosylation with > 80% of glycans found that these sites containing core fucose (Fig. 4A). The HEK Expi293F expressed protein showed fewer *N*-sites with over 80% core fucosylation, at N74, N717 and N1134 (Fig. 4B). In both proteins the N1134 site appeared to show greater than 90% fucosylation in the glycans detected. It should, however, be noted that for both HEK Expi293F and CHO-S derived spike proteins the high relative proportion of fucosylation was influenced by lower variety of glycan species present at some *N*-sites. Overall, the similarity in fucosylation levels between the CHO-S and HEK Expi293F expressed proteins suggests that the host cell line would have little effect on fucose-mediated binding and interactions of the protein.

CHO-S expressed spike protein exhibited a higher presence of sialic acid residues across all *N*-sites (55% for CHO-S vs. 34% for HEK Expi293F, Fig. 4C). This is consistent with existing studies comparing other CHO and HEK derived proteins where most glycans from both systems were found to exhibit core fucosylation, but sialylation was found to be more prevalent in CHO expressed samples [28, 29, 34, 35, 41]. However, our data shows a higher relative proportion of sialylated glycans than has been previously reported with either CHO or HEK expressed spike protein, where values for HEK sialylation have varied around 15 to 20%, and for CHO were around 40% [7, 25, 45]. Another comparison of sialylation in CHO-derived spike protein has demonstrated that the mammalian expression system produced more sialic acid content than its HEK-expressed counterpart, though only across 19–21 of the known *N*-glycosites [45]. Comparative sialylation levels between HEK and CHO derived S1 subunits appear to be considerably lower, with both having less than 10% sialic acid content [46]. However, differences in sialic acid content between equivalent *N*-sites in S1 and full length HEK-derived spike protein have been observed, suggesting that glycan modification of the subunits alone differs from that of the trimeric full length protein [25]. Additionally, evidence exists suggesting that the expression media used can also influence levels of sialylation on recombinant spike protein [46].

For the HEK Expi293F produced spike protein the highest relative proportions of sialic acid were observed at N343 and N603 (Fig. 4B), while for CHO-S high levels of sialylation were found to occur at N17, N149, N165, N603, N616, N657 (Fig. 4C). At three key functional sites (N74, N165 and N616) the CHO-S expressed protein contained more sialylated glycans than its HEK Expi293F counterpart. High levels of sialylation in the key N343 RBD glycosite has been observed previously in both HEK and CHO derived spike, however the exact distribution of sialic acid appears to differ between studies, and again between the S1 subunit and full length protein [25, 45, 46]. It is likely the assignment of these charged residues in glycan profiling is highly influenced by the sensitivity of the analysis methods used, as well as by the variation in stabilising alterations used for these recombinant constructs.

The presence and degree of sialylation is known to affect the biological activity and active half-life of glycosylated proteins [28–30], thus these changes in sialylation, both across the protein and at individual glycosites, could have a measurable impact on functionality. A recent study has demonstrated the role of sialic acid containing glycans on the cell surface in facilitating the binding and entry of the SARS-CoV-2 virus into hACE2 expressing cells [14]. This has two potential implications for drug development: firstly that highly sialylated CHO produced spike proteins for antibody based COVID 19 therapies may not be sufficiently effective to produce viable drugs, and secondly that alterations to sialylation on the wild type protein during infection, through natural mutation or therapeutic intervention, could affect the infectivity of the virus.

### Presence of Non-Human Sugar Moieties

CHO cells have been previously found to produce potentially immunogenic residues such as *N*-glycolylneuraminic acid (NGNA) and $\alpha$-1, 3-galactose. Up to 2% of circulating human polyclonal antibodies are directed against residues like these, and so could cause undesirable immune response or fast clearance of any recombinant protein exhibiting them [51–54]. In this study, the presence of *N*-glycolylneuraminic acid (NGNA type sialic acid) was found to be unique to the CHO-S derived protein. In the HEK Expi293F derived protein only *N*-acetylneuraminic acid (NANA) was found present in terminally sialylated glycans. While NANA was found to be present in the majority of terminally sialylated glycans in CHO-S (98.78% Supplementary Table S2), the non-human NGNA species was present in 9% of all terminal sialylation found (Supplementary Table S2).

NGNA is a hydroxylated form of *N*-acetylneuraminic acid created by the cytidine monophosphate *N*-acetylneuraminic acid hydroxylase (CMAH) enzyme, which is absent in human cells due to an irreversible mutation in the CMAH gene [55, 56]. Studies have shown that human consumption of mammalian-derived products results in the uptake of NGNA-containing glycoproteins through micropinocytosis [57, 58]. These epitopes are then metabolically incorporated into newly synthesised glycans, which results in the generation of complex anti-NGNA antibodies known as “Xeno-autoantibodies” [59–62]. Glycans containing NGNA are recognised by polyclonal anti-NGNA IgG, IgM and IgA antibodies that make up 0.1–0.2% of the total circulating antibodies in human serum [63–67]. For vaccine development, presentation of a spike protein antigen exhibiting non-human or potentially immunogenic glycans could result in generation of neutralising antibodies against viral epitopes that do not confer future protection against the naturally occurring SARS-CoV-2 virus [41]. Additionally NGNA has been seen to accumulate in certain pathological diseases, including cancer [68–70], and may exacerbate certain chronic inflammatory conditions such as cardiovascular disease [61, 71, 72].

**Fig. 5:**
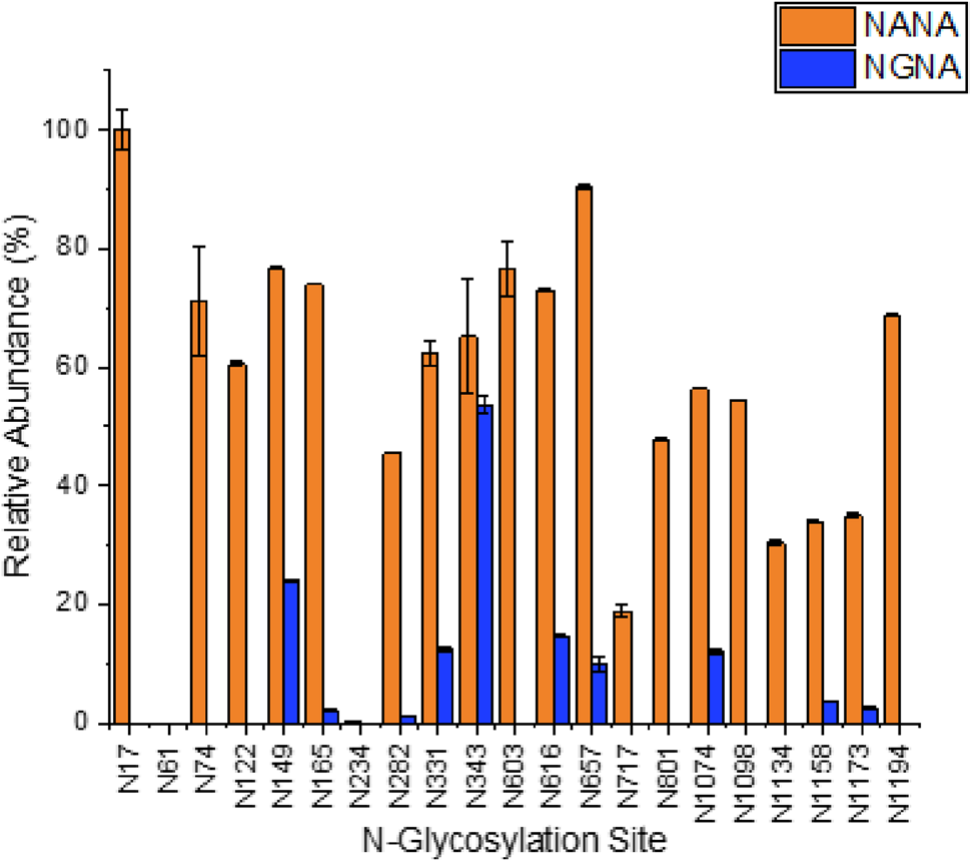
Distribution of NANA and NGNA residues in glycans across each of the 21 high confidence *N*-glycosylation sites in CHO-S derived spike protein

Figure 5 shows the distribution of NGNA and NANA residues in all glycans found at each *N*-glycosylation site. While NANA sialylation was found at 21 out of the 22 *N*-glycosites (including N706, shown only in Supplementary Table S2), NGNA was only present at 10 of the sites. Compared to NANA, the non-human sialic acid moieties made up a relatively small proportion of the terminal residues at each site. The sites with the highest relative proportion of NGNA residues were N149 and N343 (24.01% and 53.73%, respectively, of glycans present at that site). N343 has been demonstrated as a site important for mediating viral-host interaction and binding. However, the relative proportion of NGNA containing glycans at N343 was only reflective of 0.05% of the total glycans found across the whole protein. CHO cells have previously been characterised as producing similarly small amounts of NGNA, and this has been observed at the same N343 site on another CHO expressed spike protein [25]. It is important to note that, even with these reported differences in glycosylation and the potential for immunogenic residue inclusion, CHO cells have been proven to be a safe manufacturing platform for human therapeutics for the past three decades [40].

### Profiling Individual Glycan Species

**Fig. 6:**
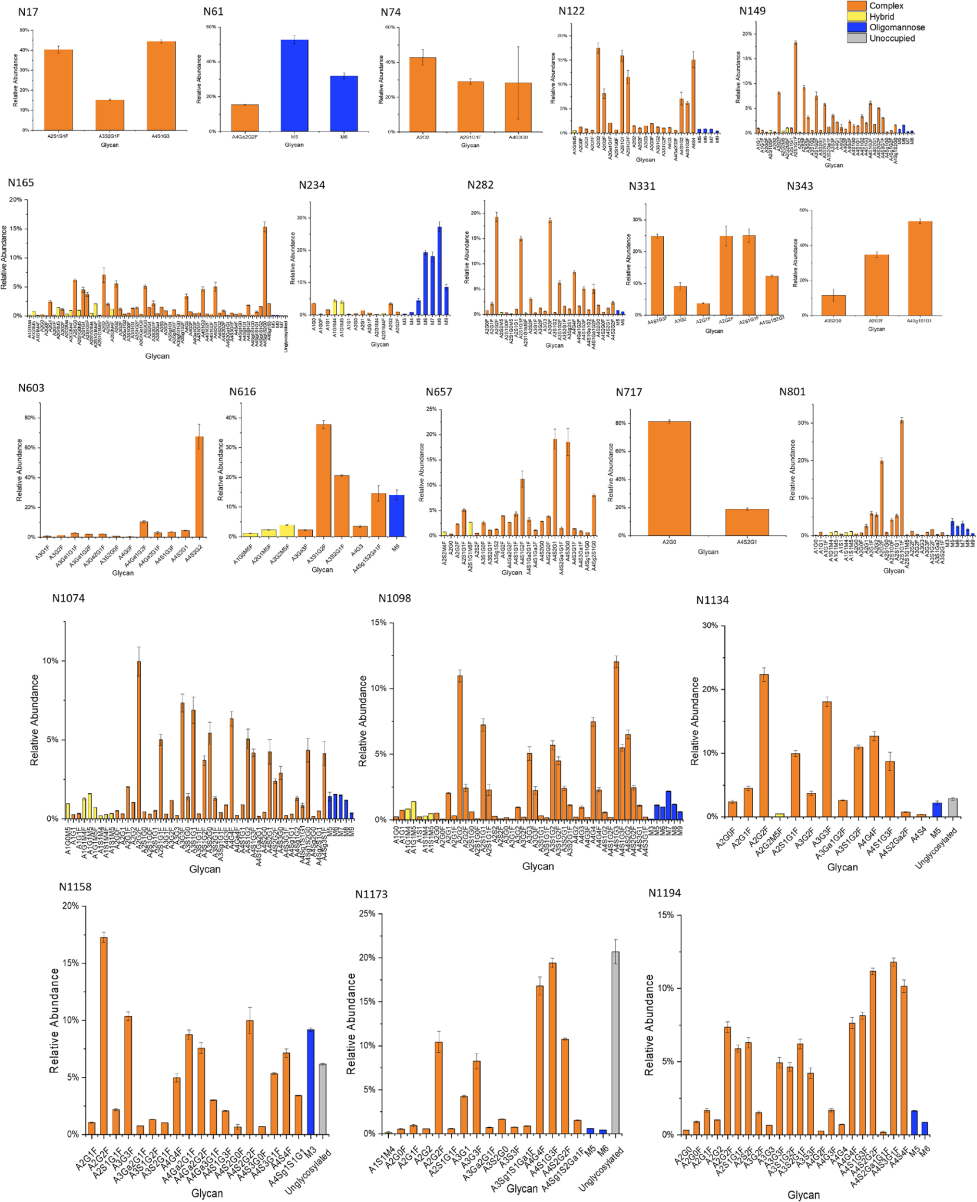
Identification of individual glycan species across 21 *N*-glycosylation sites in CHO-S derived spike protein

**Fig. 7:**
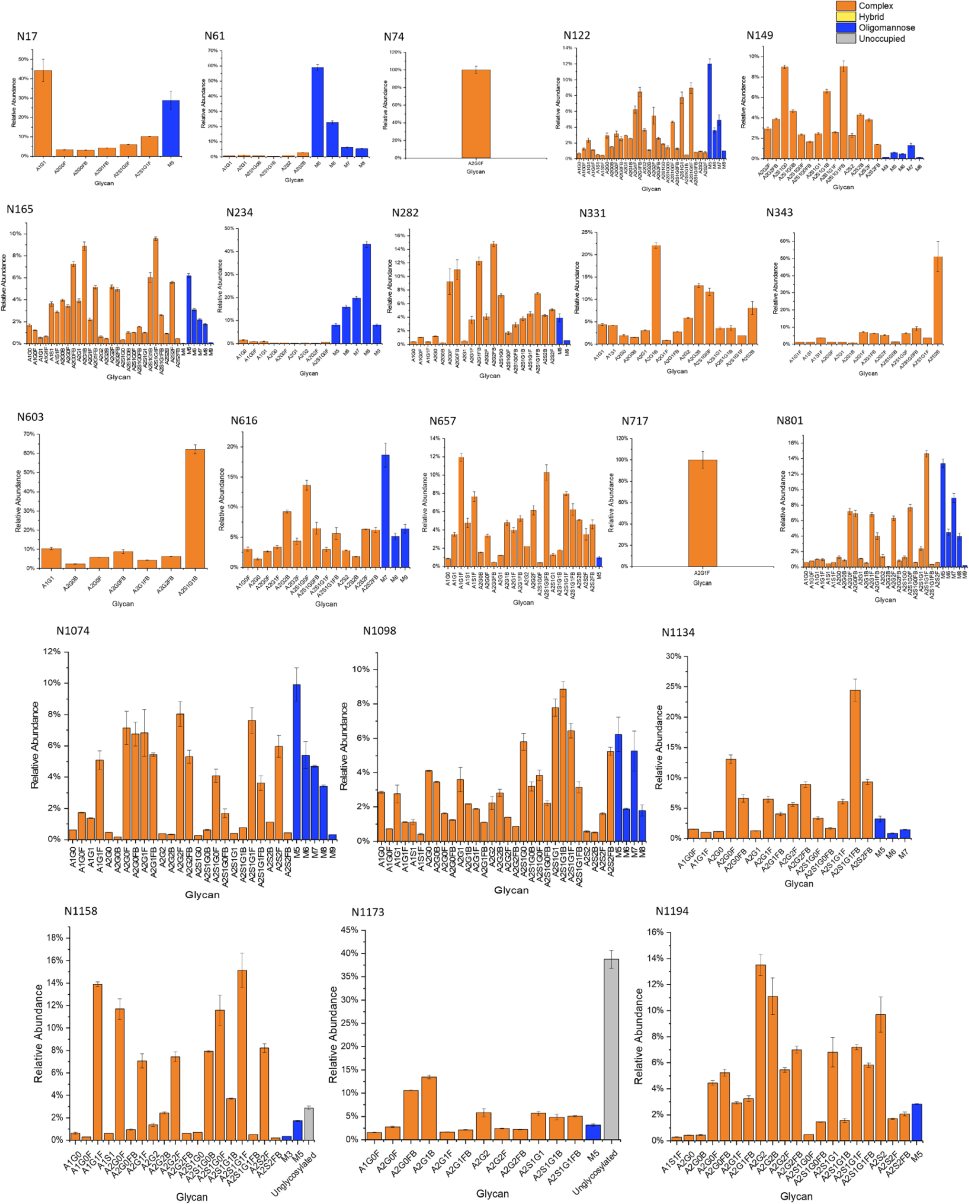
Identification of individual glycan species across 21 *N*-glycosylation sites in HEK Expi293F derived spike protein

More in depth analysis was then performed to identify and compile all the individual species of glycan found to be present at each *N*-glycosylation site. The relative abundance of each individual glycan found was calculated relative to the total glycan population (Supplementary Table S1). The glycoprofile of each *N*-site is shown in Figs. 6 and 7. The total relative abundance of all individual species was also calculated, and the most abundant glycans across the whole protein were identified (Table 1).

**Table 1:** The most abundant glycans found across all sites in CHO-S and HEK Expi293F expressed spike proteins

CHO-S		HEK Expi293F	
Glycan	Relative abundance (%)	Glycan	Relative abundance (%)
A2S1G1F	11	M5	8.90
A2G2F	9	A2S1G1F	7.91

Both spike proteins exhibited a lower variety of glycan species on S1 subunit *N*-glycosites located on the N-terminal side of the thrombin cleavage site. Conversely, the number of individual species found on the S2 subunit *N*-sites was generally higher (Figs. 6,  7). For the HEK Expi293F expressed spike protein (Fig. 7) a similar overall profile was found compared to previous studies [7], though some differences in the exact species and proportions of oligomannose glycans was observed. At the three key *N*-sites that have been previously identified as regulators of the RBD-hACE2 binding interaction (N165, N343 and N616) the HEK Expi293F expressed spike protein seemed to exhibit the greater variety of glycan species than the CHO-S expressed counterpart. A similar comparison was made by Wang et al., where the CHO derived spike protein was shown to exhibit fewer individual glycan species at N343 and N616 than a HEK derived equivalent full length protein [45]. However, our data has discovered the presence of more individual glycan species at the N165 and N343 key sites than has been previously reported in site-specific analysis of HEK derived spike protein [7, 25, 45].

The two most abundant glycans found on the CHO-S expressed spike protein were complex type, biantennary species exhibiting core fucosylation and terminal galactose on one or both branches (Table 1). For the HEK Expi293F expressed protein the most abundant glycan was found to be an oligomannose type glycan, M5, with the second most abundant being the same sialylated biantennary glycan (A2S1G1F) that was found to be the most abundant on the CHO-S protein. Again this is consistent with previous literature where the most prevalent glycans were identified as biantennary, core fucosylated, complex type species for both HEK and CHO expressed proteins [28, 34]. Previous glycoprofiling of HEK expressed spike protein by other parties have also found M5 to be a most prevalent glycan, so this may be a feature specific to this viral glycoprotein and not other mammalian, recombinant glycoproteins used in this type of comparative study [7, 25, 45, 49].

## Conclusion

Our analysis has confirmed that the spike protein construct expressed in HEK Expi293F cells exhibited a similar glycosylation profile to HEK-expressed spike proteins manufactured and analysed in other studies. By contrast, substantial differences were observed when the construct was expressed in CHO-S cells.

The findings from this study suggest that considerable changes occur to the glycosylation profile when this spike protein construct is expressed in the mammalian CHO-S system, rather than the human HEK293 cell line, Expi293F. However, further testing would be required to establish the exact impact of an altered glycopattern on the functionality of recombinant spike protein produced in CHO-S cells, and thus on its potential uses for drug development. Our data informs Quality by Design and mass spectrometry based monitoring of specific N glycosylation sites.

## Supplementary Information

Below is the link to the electronic supplementary material.

**Supplementary Table S1** Site specific analysis of glycan species present on CHO-S and HEK Expi293F derived spike protein.(xlsx 94KB)

**Supplementary Table S2** Table of glycans containing core fucose, *N*-acetylneuraminic acid and/or *N*-glycolylneuraminic acid by *N*-glycosylation site on the CHO-S derived spike protein. (xlsx 13KB)

**Supplementary Table S3** *N*-glycosylation site coverage and breakdown of individual glycan species found on CHO-S and HEK Expi293F derived Spike protein using either trypsin or GluC enzymatic digestion. (xlsx 218KB)

**Supplementary Table S4** Summary of individual glycan species found on CHO-S and HEK Expi293F derived spike protein, from pooled data generated by trypsin and GluC enzymatic digestion. (xlsx 135KB)

**Supplementary Table S5** Tables of glycan species found at *N*-glycosylation site N706 in CHO-S and HEK Expi293F derived spike protein (xlsx 132KB)

## Data Availability

The mass spectrometry proteomics data have been deposited to the ProteomeXchange Consortium via the PRIDE Partner Repository with the Dataset Identifier PXD054718.
